# Evaluating Clinical Pharmacy Services in Ministry of Health Hospitals in Al-Baha Region of Saudi Arabia, a Three-Year Retrospective Analysis

**DOI:** 10.3390/healthcare14172689

**Published:** 2026-08-24

**Authors:** Saleh Alghamdi

**Affiliations:** Department of Clinical Pharmacy, Faculty of Pharmacy, Al-Baha University, Al-Baha 65779, Saudi Arabia; saleh.alghamdi@bu.edu.sa

**Keywords:** clinical pharmacy, pharmacists, Ministry of Health, medication safety, Saudi Arabia

## Abstract

**Highlights:**

**What are the main findings?**

- Active clinical pharmacist interventions accounted for 61.8% of all documented forms over the three-year period, concentrated in adult ICUs (74.1%) and records involving patients aged 65 years or older (61.1%); among the 1077 active interventions with a documented physician decision, 99.4% were accepted. Active interventions were also highly concentrated at one of the two study hospitals (99.6%).
- ICU placement independently predicted therapy optimization (OR 2.64, *p* < 0.001) but was protective against dosage-regimen errors (OR 0.30, *p* < 0.001), showing that intervention type is driven by clinical setting rather than patient demographics.

**What are the implications of the main findings?**

- Peripheral Saudi health clusters can sustain high-density clinical pharmacy surveillance once electronic tracking and standardized documentation are in place, supporting decentralization of these services beyond tertiary centers.
- The strong physician acceptance rate and setting-dependent intervention patterns argue for expanding critical care pharmacist staffing and postgraduate residency placements in regional hospitals such as Al-Baha.

**Abstract:**

**Background:** Clinical pharmacists play a crucial role in identifying and resolving drug-related problems (DRPs) in hospitals. However, there is limited empirical evidence concerning their effect on service organizations in smaller regional facilities in Saudi Arabia. **Methods:** A retrospective serial cross-sectional study was conducted using three years of administrative data (2023–2025) from two public hospitals in the Al-Baha region. A total of 1812 different database records were identified after full-scale data cleaning, validation, and deduplication. The statistical framework included chi-square test of independence, pairwise two-proportions Z-test, three multivariate binary logistic regression models, Cramer’s V association analysis, and Ward’s hierarchical cluster modeling. **Results:** Active intervention rates significantly increased from 48.9% in 2023 to 72.4% in 2024 (z = −8.58, *p* < 0.001), stabilizing at 69.6% throughout 2025 (*p* < 0.001 versus the 2023 baseline), because 2023 data came from a different source system than 2024 to 2025 data, this pattern is reported descriptively rather as a programmatic integration. The most frequent clinical actions addressed therapy optimization (28.4%), inappropriate dosage regimens (23.6%), and missing drug orders (18.8%). Most entries recorded were for adult intensive care units (ICUs) (74.1%), and most involved patients aged 65 years or older (61.1% of records). ICU setting was independently associated with the intervention being classified as therapy optimization (adjusted odds ratio [aOR] 2.64, *p* < 0.001), but it was an inverse predictor of dosage-error tracking (OR 0.30, *p* < 0.001). **Conclusions:** This is among the first serial cross-sectional studies to build an evidence-based framework describing clinical pharmacy practices in the Al-Baha region, showing pharmacists’ roles and prioritizing advanced clinical training programs, reinforcing the need for critical analysis of health cluster policies across peripheral Saudi networks.

## 1. Introduction

The institutional healthcare system has experienced tremendous paradigm shifts in the past decades, especially regarding the provision of clinical pharmacy services. The transformation is from a simple dispensing task, focusing on compounding, stock control and mechanical retrieval of pharmaceuticals, toward a direct, patient-oriented clinical care model. Modern clinical pharmacy is based on clinically trained pharmacists integrated into multidisciplinary teams, engaging with other team members, monitoring patients at bedside, attending patient ward rounds and making real-time clinical decisions. Internationally, growing evidence shows that routinely involving clinical pharmacists in ward round reviews leads to a reduction in the rate of prescribing errors, curtails the number of severe ADRs and helps in the optimization of drug regimens. Furthermore, hospital length of stay (LOS) and preventable readmission metrics are also reduced [1,2,3,4]. The ICU is a critical environment where polypharmacy and altered pharmacokinetics, including changes in volume of distribution, renal clearance, and the use of narrow-therapeutic-index (NTI) drugs, increase risk for critically ill patients, so pharmacist-initiated surveillance and interprofessional collaboration around these parameters can be lifesaving [3,5,6]. Under the Saudi Vision 2030 Health Sector Transformation Program, Saudi Arabia has expanded its national clinical pharmacy infrastructure by deploying institutionalized advanced post-graduate residency training programs to train and develop specialized practitioners [7,8,9]. On the other hand, the Ministry of Health (MOH) deployed a centralized national electronic platform to log clinical interventions in real time [10]. While published evaluations confirm the clinical and economic value of these services in major tertiary centers within Riyadh and Jeddah, smaller secondary regional referral hospitals, such as Al-Baha, remain largely unstudied [11,12,13,14,15]. The Al-Baha region is in the southwestern highlands, with a majority of the rural population served by secondary public facilities [16,17]. Clinical pharmacy services have operated in health cluster hospitals for several years; however, the service quality has been subject to limited objective evaluation or auditing due to the absence of local longitudinal data [18]. The present study addresses this gap by conducting a three-year empirical audit of the practice patterns‚ clinical outcomes‚ and workplace determinants of clinical pharmacy interventions in MOH hospitals in the Al-Baha healthcare cluster.

This study undertakes a serial cross-sectional assessment of secondary-care public facilities in Al-Baha, describing clinical pharmacy service activity between 2023 and 2025. Descriptive analysis establishes the basic characteristics of the cohort, and multivariable and association-based statistical methods are then applied to identify patient-level and setting-level factors linked to core pharmacist actions. The aim is to provide an empirical baseline for future evaluations that is useful for healthcare providers, pharmacy services, hospital administrators, and health authorities seeking to optimize pharmaceutical care delivery in Al-Baha and comparable regional networks.

## 2. Materials and Methods

### 2.1. Study Design and Setting

Data were obtained from the MOH-maintained electronic database of the Al-Baha health cluster for a period of 3 years (1 January 2023 to 31 December 2025). Two hospitals were included: Prince Meshari bin Saud Hospital in Baljurashi (a 330-bed secondary health care center, with multi-professional critical care facilities) and King Fahad Referral Hospital (350 beds) in Al-Baha. Both sites have well-established adult ICU and internal medicine units where structured clinical ward rounds are held daily. An outpatient ambulatory anticoagulation service is available at each institution, with a fully integrated clinical pharmacy department at each site. A retrospective serial cross-sectional design was used; each study year (2023, 2024, 2025) contributes an independent annual extraction of documentation forms from the MOH registry, rather than a probability sample or repeated observations on the same patients, so the study describes serial cross-sectional activity based on routinely generated administrative records rather than a longitudinal cohort.

### 2.2. Data Source and Collection

The primary data source was the MOH Centralized Clinical Pharmacy documentation platform, where institutional clinical pharmacists recorded patient care activities in real-time, using standardized digital forms. Data for the year 2023 were collected from the regional administrative registry, whereas for years 2024 to 2025, national electronic platforms were utilized. Data were filtered to include only Al-Baha healthcare cluster hospitals. All the data was extracted in Microsoft Excel ^®^ (Office 365, Microsoft Corporation, Redmond, WA, USA) sheets for further analysis. A direct comparison of column labels and category values between the two source files was performed even without a surviving data dictionary; the files themselves serve as the record. Seventy-seven of the 2023 file’s 84 columns are present with identical labels in the 2024–2025 file. The seven 2023-only columns comprise two patient-education action sub-fields (populated for 12 records), two drug-unavailability sub-fields (8 records), and three HbA1c monitoring fields (0 records populated). Core analytical fields, encounter category, gender, age band, and form type, carry identical category values across both systems. The Type of intervention field showed four label variants in the 2023 file that were consolidated or renamed by 2024, “Providing information” (n = 12) maps to “Providing information, counselling/education”, “Unavailable/Non formulary” (n = 8) and one typographic variant (n = 1) map to “Recommend alternative medication in case of unavailability”, and “writing parenteral nutrition order (PN)” (n = 5) has no 2024–2025 equivalent, as no parenteral nutrition records appear in either year. This harmonization used the same deterministic, priority-ordered keyword ruleset described in Section 2.4 (Supplementary Table S1), which documents the mapping of each 2023-specific label to its 2024–2025 equivalent explicitly. The encounter category field, the primary variable distinguishing active interventions from file-review visits, ambulatory follow-ups, and reconciliation forms, carries identical values in both systems, so the proportion of forms classified as active interventions reflects the same operational definition in all three years. What changed structurally was the specialty field: the 2023 system recorded ambulatory and anticoagulant clinic encounters under a specialty value, while the 2024–2025 system recorded these as a null in the encounter category field instead—a platform redesign rather than a clinical change. Whether mandatory field requirements, form sequencing, or documentation guidance differed between the two systems cannot be determined from the files alone, as no platform administration documentation survives for the discontinued 2023 registry; this remains the genuine limitation. Physician-acceptance responses are present in the source data for all three years, though completeness differs (94.4% of 2023 records, 97.8% of 2024, 99.1% of 2025 have a documented response). The cross-tabulation of year by hospital by clinical setting is provided as Supplementary Table S2.

### 2.3. Selection Criteria and Sample Harmonization

Records were extracted from three source files covering the Al-Baha region: a 2023 regional file (831 rows), a 2024–2025 regional file (861 rows), and a national dataset (71,242 rows spanning 20 regions). The national dataset was first filtered to Al-Baha records (n = 1108), from which 861 records already present in the 2024–2025 regional file were identified by exact-timestamp matching and removed, yielding 247 unique 2025 records not otherwise captured. All verified clinical intervention forms recorded during the study period were eligible. Specialized training records and educational lecture records were identified by the platform-assigned form-type field and excluded within each source file before concatenation, 54 training and 17 lecture records from the 2023 regional file (831 to 760), 34 training and 9 lecture records from the 2024–2025 regional file (861 to 818), and 6 training and 7 lecture records from the unique 2025 subset of the national file (247 to 234), a total of 94 training and 33 lecture records excluded. The three cleaned sources were then concatenated (760 + 818 + 234 = 1812) and checked for exact-timestamp duplicates, of which none were found, yielding the analytic total of 1812 entry forms. Of the 1812 forms, 1120 were active clinical interventions, 145 were file reviews with no drug-related problem identified, 543 were ambulatory anticoagulation or outpatient follow-up encounters, and 4 were medication reconciliation forms, forming the primary analytic cohort described in Table 1. The full extraction and exclusion pipeline is shown in Supplementary Figure S1.

### 2.4. Classification and Recoding of Variables

Free-text clinical narrative strings and raw intervention data were mapped to eleven mutually exclusive domains, matching the categories reported in Table 2, therapy optimization, dosage regimen error, missing drug order, electrolyte correction, ADR or drug interaction, inappropriate drug selection, no indication (a drug ordered without a supporting diagnosis), drug unavailability, patient education, therapeutic drug monitoring (TDM), and parenteral nutrition support. Classification was performed using automated keyword matching applied to the raw intervention-type strings entered by pharmacists into the MOH digital platform, without manual review of individual records and without a second independent coder. A deterministic, priority-ordered ruleset of eleven patterns was applied case-insensitively, the first matching rule determined category assignment, ensuring mutual exclusivity. The full ruleset is provided as Supplementary Table S1. No inter-rater reliability coefficient was calculated, as there was no human coding process against which to assess agreement. Where a single form contained more than one documented issue, the platform captured only the primary intervention type in the field analyzed. Records matching no rule were assigned to an “Other” category for manual review, no such records were identified in the present dataset. Platform terminology evolved between 2023 and 2024 following a system update such as, “guideline deviation” refers to the platform category Inappropriate Drug Selection (“inappropriate drug selection against guideline or recommendation”), “failure to order an electrolyte” corresponds to Electrolyte Correction (“correct the cause of electrolyte disturbance”), and “structured patient counselling” corresponds to Patient Education (“providing information, counselling or education”). All category labels used in Table 2 reflect platform terminology, mapped explicitly to the classification rules in Supplementary Table S1. Patient age was entered into the regression models as a continuous ordinal score (0 to 7, one point per successive age band from under 1 year to 65 years and above), so the reported odds ratio reflects the change in odds per one-category increase in age band rather than a comparison against a discrete reference group. A sensitivity analysis recoding age into four unequal categorical bands (0 to 24, 25 to 54, 55 to 64, 65 and above, reference 0 to 24) is reported alongside the main models and yields the same conclusions. Missing demographic fields (gender not recorded for 4.6%, n = 52, and age not recorded for 3.7%, n = 41, of which 26 records were missing both, for 67 records missing at least one field) were managed by listwise deletion, leaving a complete-case analytic sample of n = 1053 for all three regression models. The covariates entering each model were patient age band, gender, and study year (Model 1), plus ICU setting (Models 2 and 3), hospital site, comorbidity, and free-text diagnosis were not modeled. Clinical environment was coded as ICU or non-ICU, and structured physician acceptance codes determined interprofessional collaboration. Patient file numbers, recovered from the raw source files, permitted patient-level clustering, 42 of the 1120 active-intervention records (3.8%) had no recoverable file number and could not be linked to a patient. Within the complete-case regression sample (n = 1053), 421 records shared a file number with at least one other record and 12 had no recoverable file number, each of these 12 was assigned a unique singleton cluster in the regression models described below, so it contributed independently without being assumed to share correlation with any other patient.

### 2.5. Statistical Analytics Framework

Analyses were performed using R version 4.2.2 (R Foundation for Statistical Computing, Vienna, Austria). Categorical variables and descriptive elements were reported in terms of frequencies and percentages. Pearson’s chi-square tests for independence assessed association between intervention type and clinical parameters; effect sizes were quantified with Cramér’s V. Cramér’s V coefficients were interpreted as small (0.10–0.29), moderate (0.30–0.49), or large (0.50 and above). Two proportions Z-tests compared any changes in volume of each intervention over time (year-to-year comparisons). Three multivariable models were fitted using generalized estimating equations (GEE) with a binomial family, an exchangeable working correlation structure, and robust sandwich standard errors, to determine independent predictors of core pharmacist actions while accounting for within-patient clustering. The clustering unit was the patient file number; records with no recoverable file number were each treated as an independent singleton cluster. For Model 1, demographic and temporal factors were used to predict ICU placement; for Model 2, demographic, temporal, and setting (ICU) factors were used to predict dosage-regimen-error classification; for Model 3, the same factors were used to predict therapy-optimization classification. Adjusted odds ratios (aOR) and 95% confidence intervals (CI) are reported for each model. Standard (non-clustered) binary logistic regression, assessed using Nagelkerke R^2^ and likelihood-ratio χ^2^, was also fitted as a sensitivity comparison. The analysis of large contingency tables was done by using standardized Pearson residuals to identify statistical anomalies in individual cells, with a significance value of 2.0 (absolute values > 2.0 were considered to represent a significant deviation from the expected null distribution). Hierarchical cluster modeling was performed using Ward’s minimum-variance linkage, intended to describe underlying practice patterns descriptively rather than to test the intervention type by setting association formally (see Section 4.2 for the corresponding note on the distance matrix, standardization approach, and cluster-stability validation). All inferential tests were conducted at an alpha level of 0.05 (α = 0.05).

### 2.6. Ethical Considerations

The study was conducted in accordance with the Declaration of Helsinki and approved by the scientific and research committee of the main hospital involved in this study, King Fahad Hospital in Al-Baha, Saudi Arabia (Approval NO., KFH-17052026-5).

## 3. Results

### 3.1. Overview of the Study Population and Cohort Characteristics

Data extraction resulted in a total of 1812 documentation forms across the three-year study window, contributed unevenly by the two study hospitals (Prince Meshari bin Saud Hospital, Baljurashi, 1188 forms, 65.6%, and King Fahad Hospital, Al-Baha, 624 forms, 34.4%). After data cleaning and deduplication, 1120 (61.8%) were active clinical pharmacist interventions. The remaining 692 forms were routine file reviews with no identified drug-related problem (8.0%, n = 145), ambulatory anticoagulation or outpatient clinic follow-up encounters (30.0%, n = 543), or medication reconciliation forms (0.2%, n = 4). Active interventions were themselves highly concentrated at Prince Meshari bin Saud Hospital (99.6%, n = 1116 of 1120), with only 4 active interventions recorded at King Fahad Hospital during the study period. Baseline characteristics are shown in Table 1.

**Figure 1:**
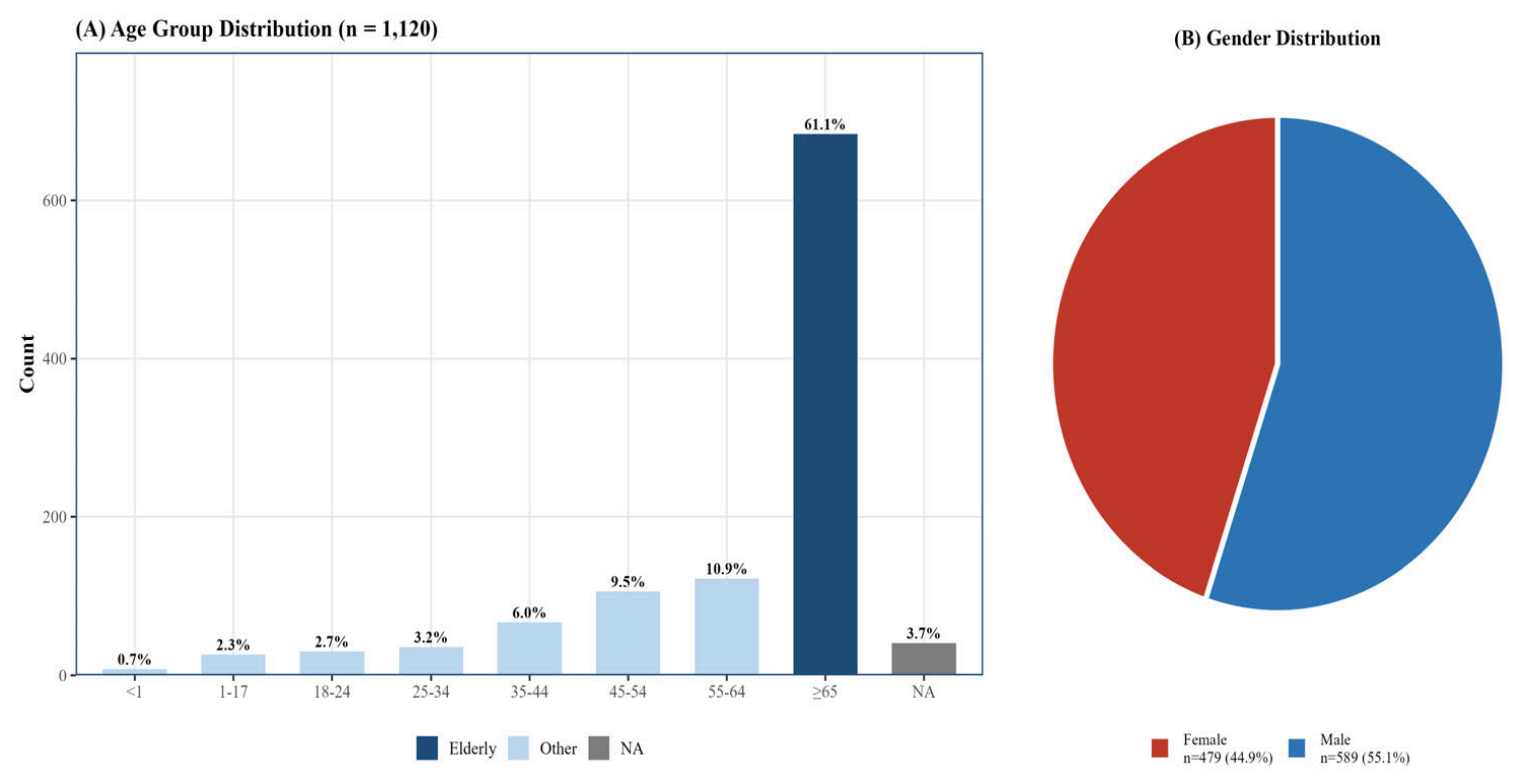
Demographic profile of patients receiving pharmacist interventions in Al-Baha hospitals (**A**) age group and (**B**) gender distribution (2023–2025, n = 1120).

Slightly more active-intervention records involved male (52.6%, n = 589) than female patients (42.8%, n = 479); gender was not recorded for 4.6% (n = 52) of records (Figure 1B). Because the dataset has no patient identifier, these are proportions of intervention records, not of unique patients, and the same patient may be represented more than once. The majority of active-intervention records involved patients aged 65 years or older (61.1%, n = 684) (Figure 1A). Total documented forms varied across the study period, from 760 in 2023 to 568 in 2024 and 484 in 2025. The proportion of each year’s documentation that was an active intervention rose from 48.9% (372 of 760) in 2023 to 72.4% (411 of 568) in 2024 (z = −8.58, *p* < 0.001), then stabilized at 69.6% (337 of 484) in 2025 (z = −7.18, *p* < 0.001 versus 2023 baseline, z = 0.974, *p* = 0.330 versus 2024), this shift coincides with the change in data source described in Section 2.2 and is reported descriptively rather than as evidence of programmatic integration. Considered as a share of the pooled active-intervention cohort rather than of each year’s own documentation, these same 372, 411, and 337 interventions correspond to 33.2%, 36.7%, and 30.1%, respectively. Baseline demographic details and institution-wise parameters are presented in Table 1.

### 3.2. Intervention Categories and Temporal Trends

**Figure 2:**
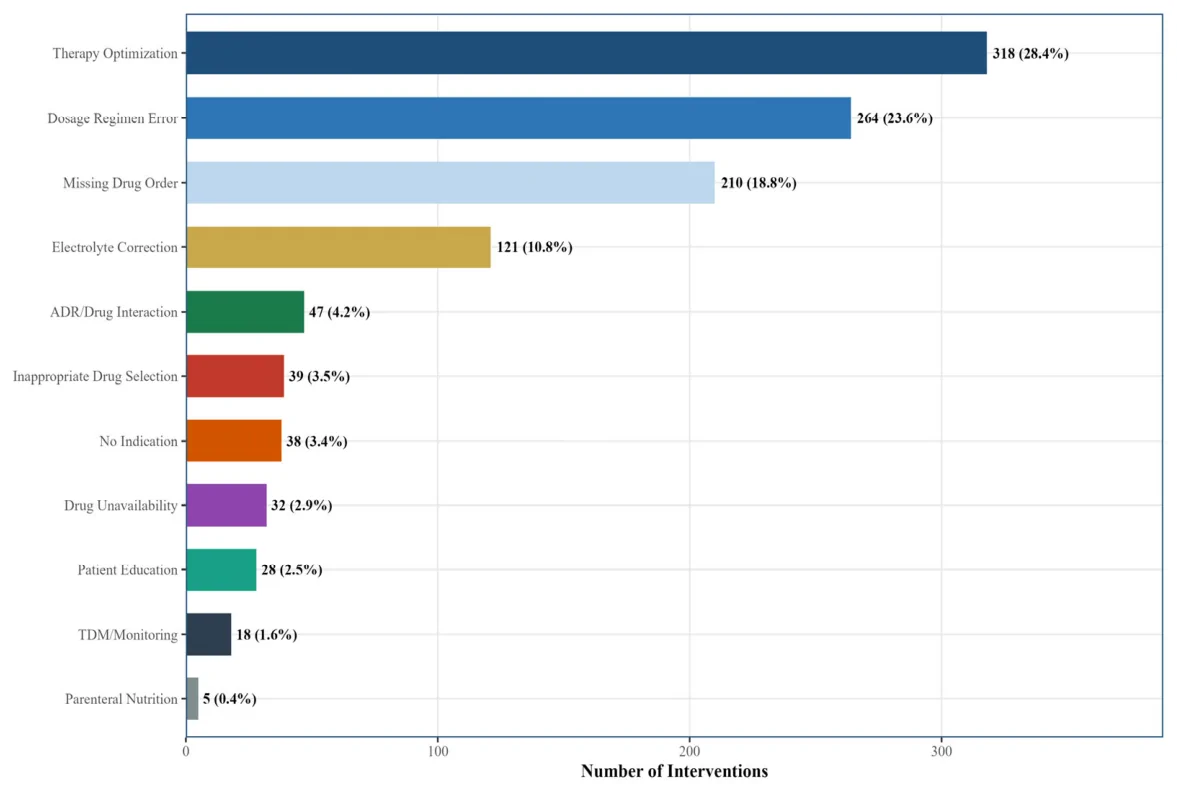
Distribution of clinical pharmacist intervention types in Al-Baha hospitals (2023–2025, n = 1120).

Therapy optimization was the primary activity across the three-year study window, accounting for 28.4% (n = 318) of active interventions (Figure 2). Inappropriate dosage regimens represented the second most prevalent drug-related problem, at 23.6% (n = 264). Within this category, route-of-administration errors were most common, followed by dose omissions and frequency errors. Missing drug orders for established medical conditions constituted 18.8% (n = 210), frequently involving omissions in guideline-directed medical therapy for heart failure (n = 19) and secondary cardiovascular protection in diabetics (n = 16).

**Figure 3:**
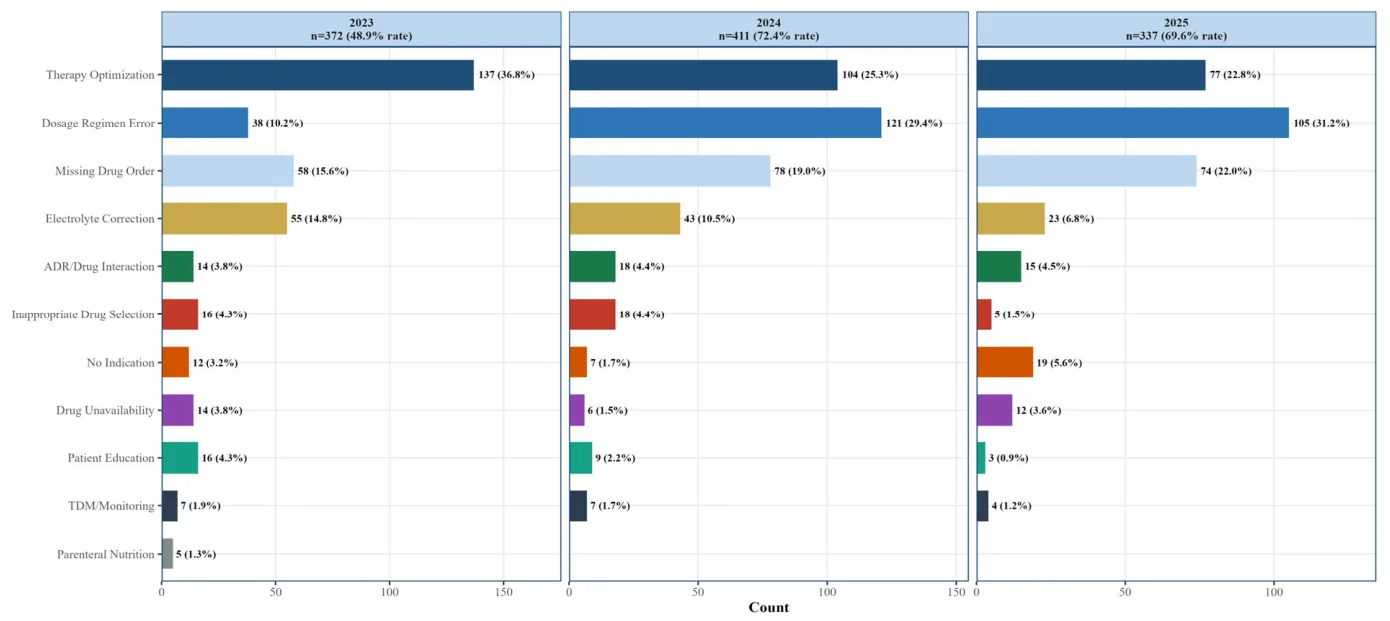
Three-year trends in clinical pharmacist intervention types in Al-Baha region hospitals (2023–2025, n = 1120).

Electrolyte corrections comprised 10.8% (n = 121) of records, showing a heavy concentration within acute resuscitation tracks for the management of potassium, magnesium, and calcium imbalances. Annual active interventions are shown in Figure 3. Year-by-year stratification revealed distinct fluctuations across the study periods, Figure 3. Active therapy optimization interventions were highest in 2023 at 36.8% (n = 137), declined to 25.3% (n = 104) in 2024, and then further adjusted to 22.8% (n = 77) in 2025. Dosage regimen corrections, by contrast, rose from 10.2% (n = 38) in 2023 to 29.4% (n = 121) in 2024, and stabilized at 31.2% (n = 105) in 2025. These year-wise operational trends are given in Table 2.

### 3.3. Institutional Settings and Clinical Specialties

**Figure 4:**
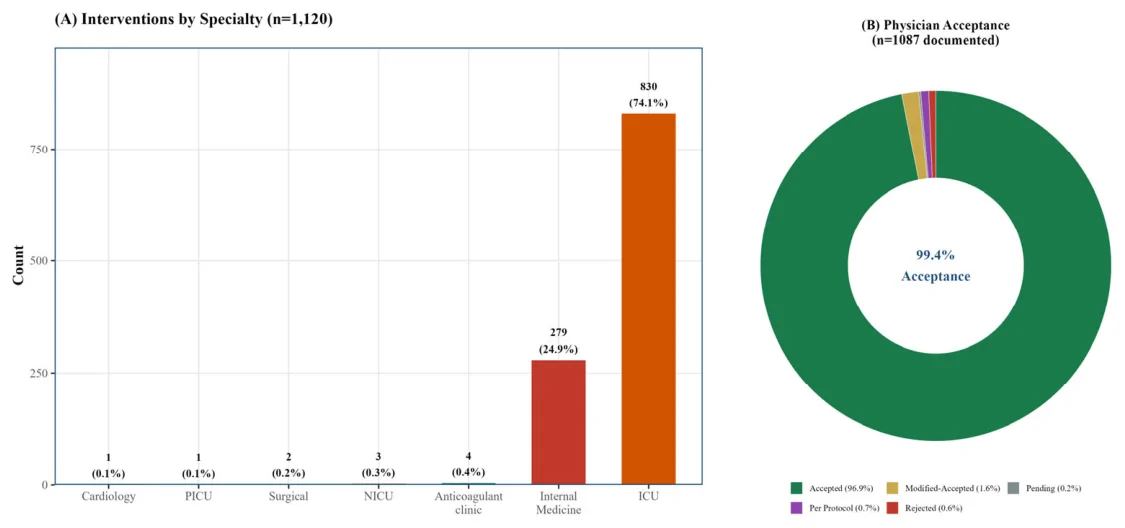
Graphical mapping of (**A**) clinical specialty distributions and (**B**) inter-professional physician acceptance rates.

Adult ICUs were the main operational area of the regional cluster with 74.1% (n = 830) of all clinical pharmacist logged interventions. The secondary operational domain was adult internal medicine medical wards with 24.9% (n = 279) entries. Fewer intervention profiles were obtained for specialized sub-acute units, such as the neonatal intensive care unit (NICU, 0.3%, n = 3), surgical wards (0.2%, n = 2), and cardiac and pediatric intensive care tracks (0.2%, n = 2) (Table 2 and Figure 4A). As noted above, 99.6% of active interventions (n = 1116 of 1120) originated from Prince Meshari bin Saud Hospital, so the specialty and outcome patterns reported here predominantly describe practice at that site. The complete breakdown of specialties is shown in Table 3. Structured physician-response data were documented for 1087 of the 1120 active interventions across all three study years; the field was blank for the remaining 33 records (2.9%). Within the 1087 documented responses, physicians accepted the recommendation without modification in 1053 cases, modified and then accepted it in 17, proceeded per an existing protocol not requiring individual physician approval in 8, left the response pending at the time of data extraction in 2, and rejected it in 7. Excluding per-protocol and pending responses, since neither represents a completed physician decision, the acceptance rate among the 1077 documented physician decisions was 99.4% (1070 of 1077) (Figure 4B).

### 3.4. Comorbidity and Pharmacotherapeutic Profiles

**Figure 5:**
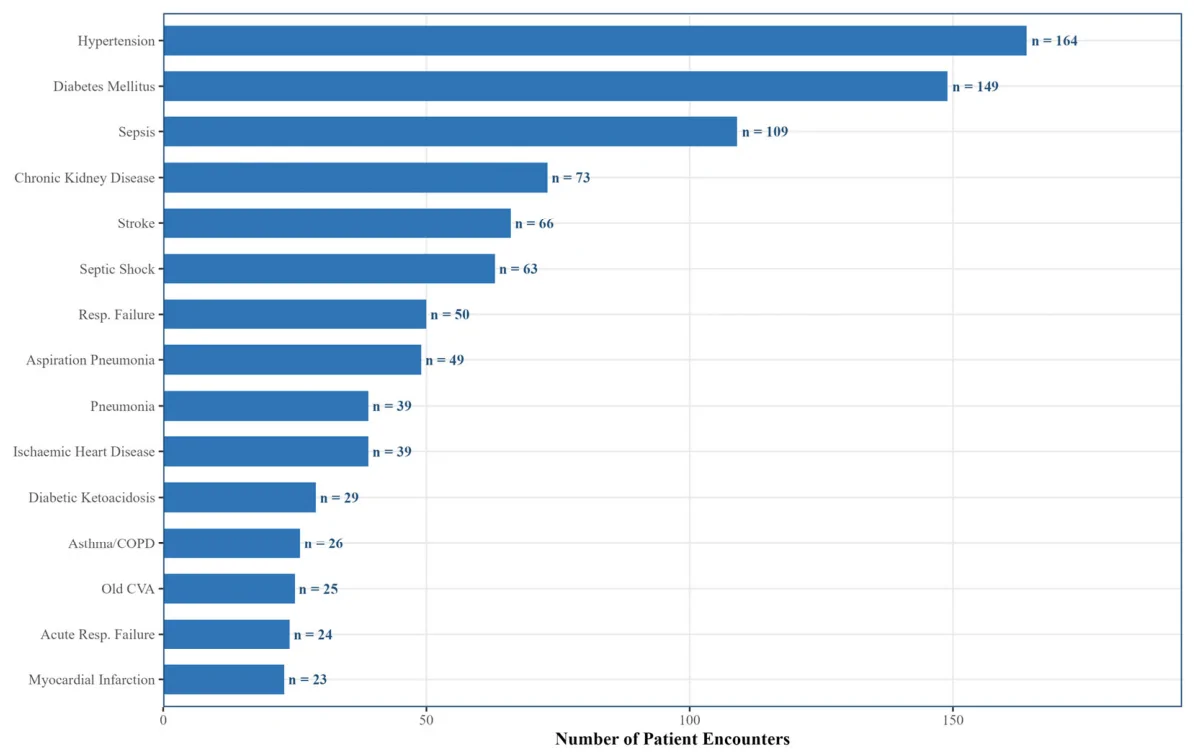
Number of active intervention records by clinical condition.

**Figure 6:**
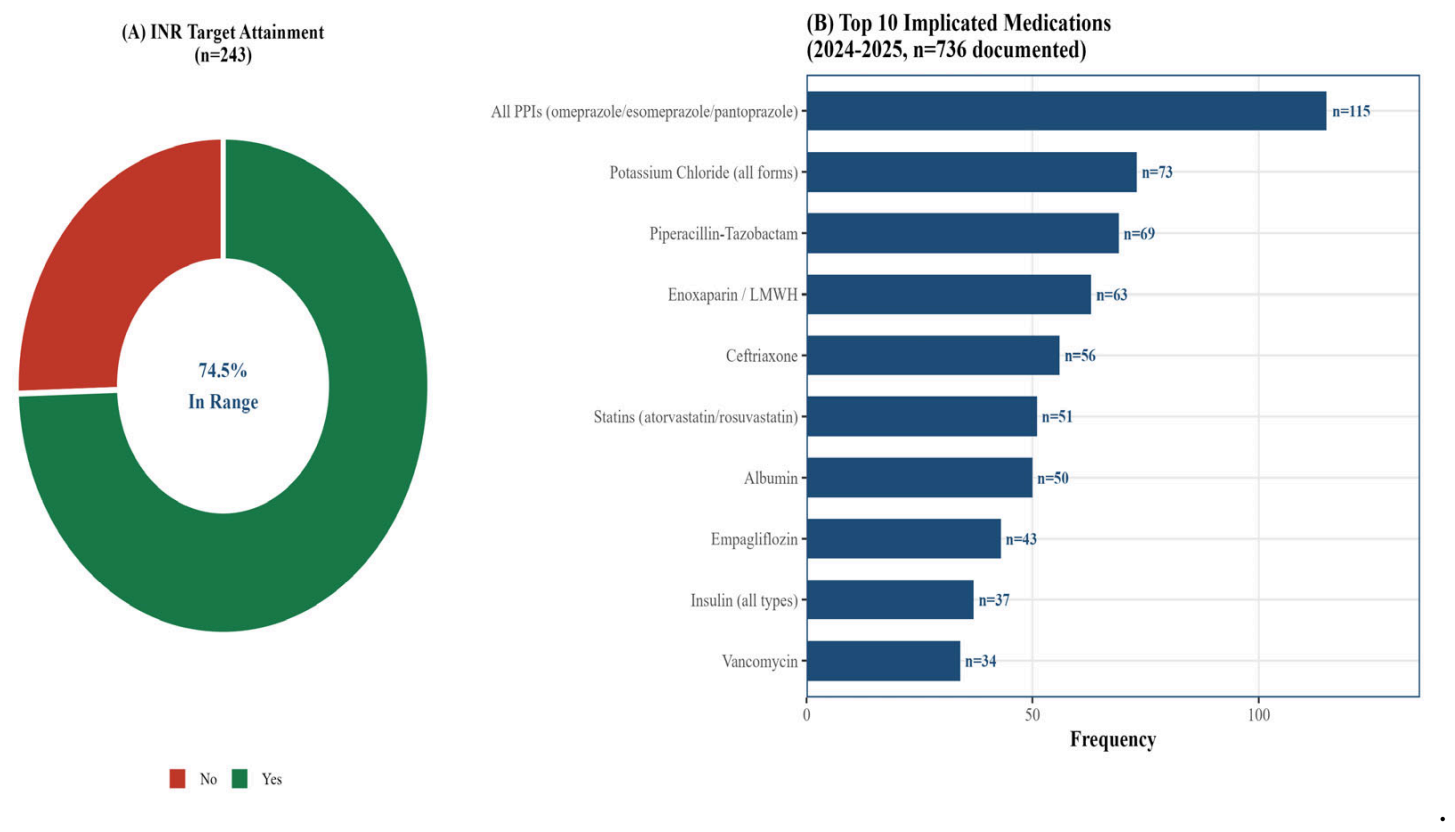
(**A**) outpatient target-INR attainment and (**B**) top 10 medications most frequently implicated in documented pharmacist interventions across all intervention types, 2024–2025 (n = 736 documented).

The primary systemic clinical diagnoses were hypertension (n = 164), type 2 diabetes mellitus (n = 149), and acute sepsis (n = 109). Shock (n = 63), acute type II respiratory failure (n = 50), and aspiration pneumonia (n = 49) were also reported. A detail of all comorbidities is illustrated in Figure 5. Medication involvement is reported here for the 2024 to 2025 subset in which drug names were captured (n = 736 documented interventions), this field was not available for 2023 records. In high-acuity critical care settings, many patients presented with septic conditions requiring antimicrobial therapy. Among named agents, proton pump inhibitors as a class (omeprazole, esomeprazole, and pantoprazole combined) were the most frequently implicated medication group (n = 115, 15.6% of documented interventions), with omeprazole alone accounting for 97 (13.2%). Potassium chloride in all forms followed (n = 73, 9.9%), then piperacillin-tazobactam (n = 69, 9.4%) and enoxaparin or other low-molecular-weight heparins combined (n = 63, 8.6%, with enoxaparin alone accounting for n = 49, 6.7%). The top 10 medications involved are presented in Figure 6B. In the ambulatory anticoagulation service, INR values within the prescribed target range were recorded at 74.5% (181 of 243) of clinic visits with a documented INR outcome (Figure 6A), this represents visit-level, not patient-level, attainment and covers 243 of the 543 ambulatory anticoagulation and outpatient follow-up encounters in the dataset, so it should be read as a description of the encounters with recorded INR data rather than as a validation of overall service stability.

### 3.5. Bivariate and Multi-Method Analysis

**Figure 7:**
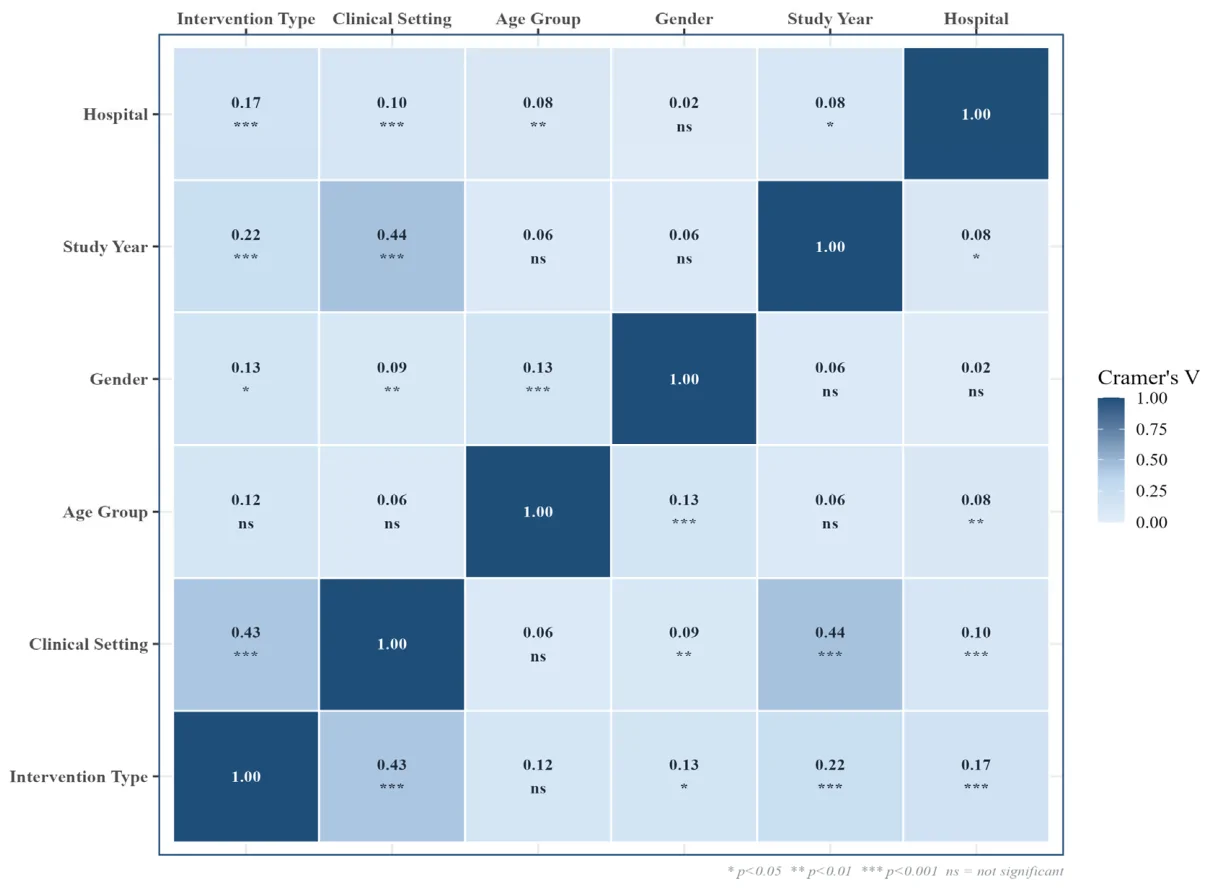
Symmetric correlation matrix of Cramér’s *V* values.

Pearson’s chi-square (χ^2^) testing was re-run directly from the analytic dataset for this revision, since the degrees of freedom in the original submission (df = 9 for the three binary comparisons, df = 18 for study year) were inconsistent with the eleven-category intervention classification used throughout the paper. The corrected tests, reported in Table 4, confirm significant associations between intervention type and each clinical parameter. A moderate to large association was found between intervention type and clinical setting (χ^2^ = 203.43, df = 10, *p* < 0.001, V = 0.426). A significant but small association was found between intervention type and patient gender (χ^2^ = 18.69, df = 10, *p* = 0.044, V = 0.132), a smaller effect than originally reported. Intervention type was not significantly associated with age group once the elderly threshold and the 41 undocumented-age records were corrected (χ^2^ = 16.36, df = 10, *p* = 0.090, V = 0.123, the originally reported χ^2^ = 44.71 had used a 55-year rather than 65-year threshold and had silently included undocumented-age records as non-elderly), and was associated with study year (χ^2^ = 108.86, df = 20, *p* < 0.001, V = 0.220). The corrected association strengths are visually mapped in the nominal association matrix (Figure 7).

### 3.6. Hierarchical Cluster Analysis

**Figure 8:**
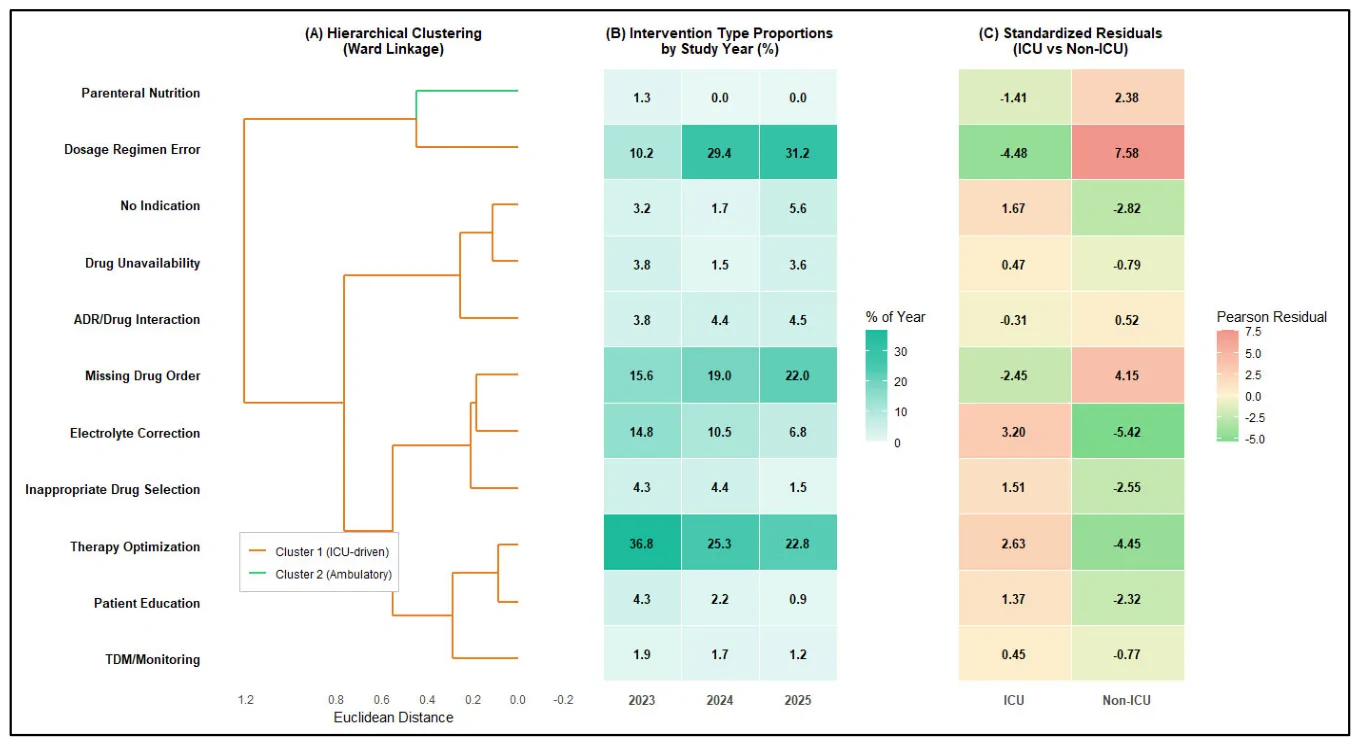
(**A**) Ward’s hierarchical cluster dendrogram, (**B**) Trend heatmap, and (**C**) standardized Pearson residuals.

Ward’s minimum-variance linkage clustering was applied descriptively to the eleven intervention categories to summarize co-occurring setting and volume patterns; it was not used as a confirmatory or inferential test of the intervention type by setting association already established by the chi-square analysis above (χ^2^ = 203.43, df = 10, *p* < 0.001, V = 0.426). Two broad clusters were visible in the resulting dendrogram (Figure 8), described below. The distance matrix (five features per intervention category—mean age band, ICU proportion, male proportion, and 2024 and 2025 proportions—with Euclidean distance and Ward’s minimum-variance linkage) and the resulting two-cluster split have been re-verified independently for this revision, but the two-cluster solution was not formally validated against alternative solutions and cluster-stability checks (e.g., bootstrap resampling) were not performed, this is noted as a limitation, and the cluster analysis is reported here as descriptive and exploratory rather than confirmatory.

#### 3.6.1. Cluster Composition

Ward’s linkage identified Cluster 1, comprising ten of the eleven intervention categories, and Cluster 2, comprising Parenteral Nutrition alone (n = 5, 0.4% of active interventions) (Figure 8A). Parenteral Nutrition separated from the other ten categories at a Euclidean distance of 1.21, distinctly higher than the next-highest merge point within Cluster 1 (0.77), reflecting its markedly lower ICU proportion (20%, versus 50–99% for every other category) and its absence from the 2024 and 2025 cohorts (all five records occurred in 2023). Within Cluster 1, the ten categories did not separate further into distinguishable sub-groups at this resolution; the ICU-versus-non-ICU differences reported via standardized Pearson residuals (Figure 8C) come from the chi-square and residual analysis in Section 3.5, not from the cluster analysis itself. When evaluated across the three years, therapy optimization was consistently the most prevalent activity within Cluster 1, at 36.8% in 2023, 25.3% in 2024, and 22.8% in 2025 (Figure 8B).

#### 3.6.2. Temporal Trend Within Cluster 1

Within Cluster 1, standardized Pearson residuals showed ICU overrepresentation in therapy optimization (+2.6) and electrolyte correction (+3.2), and non-ICU overrepresentation in missing drug order (+4.2) and dosage regimen error (+7.7) (Figure 8C). The dosage regimen errors were trending upwards during the study period (across the study years, Figure 8B), with a significant increase in the proportion of errors to 29.4% in 2024 and plateauing at 31.2% at the end of 2025.

### 3.7. Multivariable Logistic Regression

Considering the time adjustments, three GEE models accounting for within-patient clustering identified patient- and setting-level independent factors for core clinical actions. In model 1 (ICU placement), age band was positively associated with ICU tracking (OR 1.16, 95% CI 1.01–1.34, *p* = 0.036), whereas male gender was not associated (OR 0.92, 95% CI 0.70–1.22, *p* = 0.554). In model 2 (dosage error), ICU setting was an inverse predictor of dosage-error classification (OR 0.30, 95% CI 0.21–0.43, *p* < 0.001). ICU placement was the strongest positive predictor of therapy-optimization classification (OR 2.64, 95% CI 1.79–3.88, *p* < 0.001) in model 3. Age band showed no independent association with therapy-optimization classification once patient clustering was accounted for (OR 1.08, 95% CI 0.99–1.18, *p* = 0.098), a change from the uncorrected estimate reported in the original submission. All odds ratio configurations for the three models are shown in Table 5.

Across the three models, ICU setting and study year showed larger and more consistent associations with intervention classification than patient demographic factors. In Model 1 (ICU placement), age band was positively associated with ICU placement once within-patient clustering was accounted for (aOR = 1.16, 95% CI 1.01–1.34, *p* = 0.036), while male gender showed no association (aOR = 0.92, 95% CI 0.70–1.22, *p* = 0.554). In Model 2, ICU setting was associated with lower odds that a documented active intervention was classified as a dosage-regimen error rather than another category (aOR = 0.30, 95% CI 0.21–0.43, *p* < 0.001). In Model 3, ICU setting was associated with higher odds that an intervention was classified as therapy optimization rather than another category (aOR = 2.64, 95% CI 1.79–3.88, *p* < 0.001). These associations describe how documented interventions were categorized, not the rate, prevention, or clinical consequences of medication errors, which this dataset does not measure. Age band showed no independent association with either the dosage-error category (aOR = 0.97, 95% CI 0.88–1.08, *p* = 0.622) or the therapy-optimization category (aOR = 1.08, 95% CI 0.99–1.18, *p* = 0.098) once patient clustering was accounted for. All three models were fitted using generalized estimating equations with an exchangeable working correlation structure and robust standard errors, clustering on patient file number (n = 1053 records, 433 clusters.

**Figure 9:**
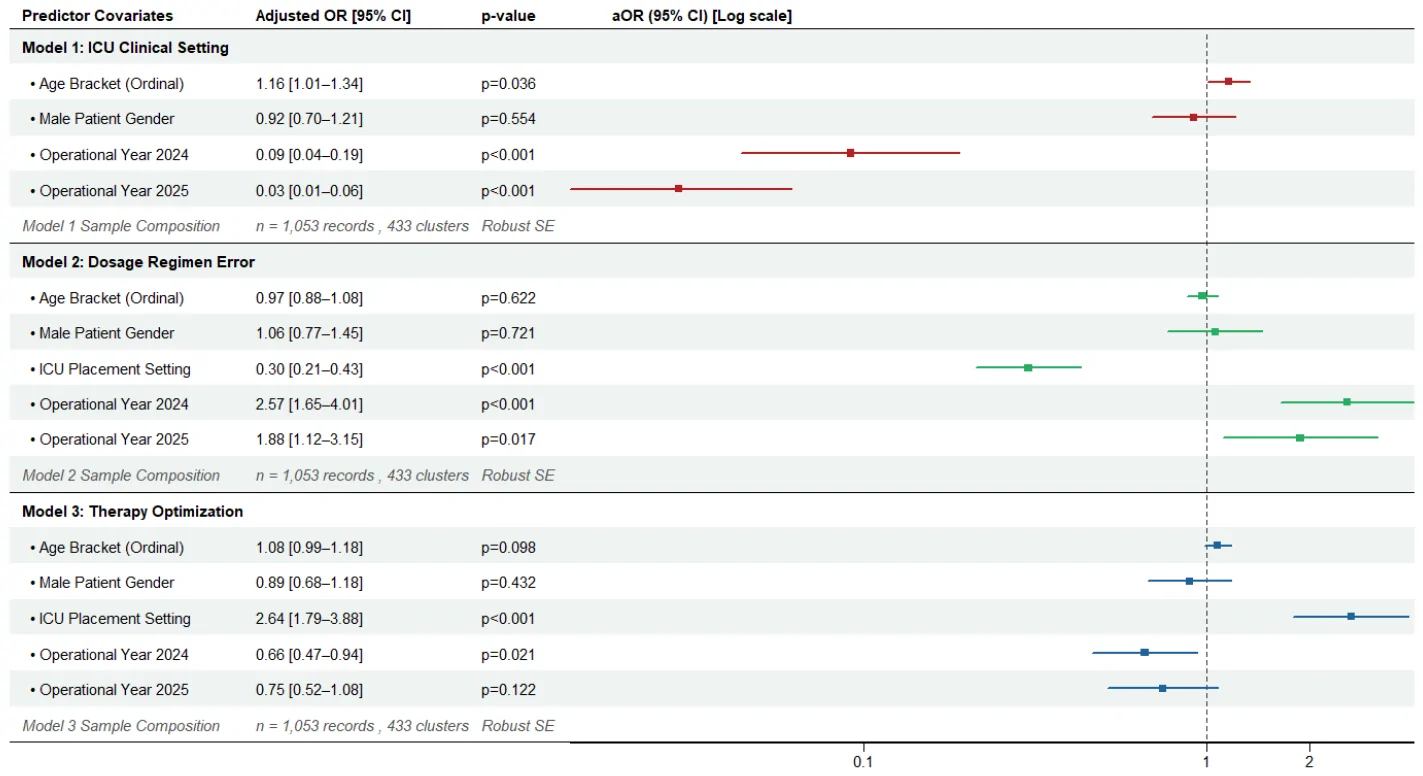
Forest plot, GEE models (exchangeable working correlation, robust standard errors) for pharmacist intervention predictors, n = 1053 records, 433 clusters. Red markers and confidence intervals correspond to Model 1, green to Model 2, and blue to Model 3. The dashed vertical line marks the null value (aOR = 1).

Figure 9 shows that documented ICU-placement odds fell markedly across the study period (aOR 0.09 in 2024, aOR 0.03 in 2025, both versus 2023), alongside a rise in dosage-error odds (aOR 2.57 in 2024, aOR 1.88 in 2025) and a temporary reduction in therapy-optimization odds (aOR 0.66 in 2024). Interpretation of these year effects is provided in the Section 4.

In summary, ICU placement was the strongest and most consistent predictor of which category a documented active intervention was assigned to, exceeding the contribution of patient age or gender in Models 2 and 3; age band was independently associated with ICU placement itself in Model 1 once within-patient clustering was accounted for. This describes how interventions were classified and recorded, not the incidence of medication errors or their clinical consequences, which this dataset does not measure. The year-on-year shifts in odds are interpreted in the Section 4.

## 4. Discussion

This is one of the first serial cross-sectional analyses of clinical pharmacy service activity in the regional context of Al-Baha, an area rarely represented in literature. The three annual samples show that documented clinical pharmacy activity was not a one-off exercise but a pattern that was low in the first year and rose and then stabilized over the following two years. The interventions were widely accepted by the prescribers and demonstrate the comprehensive training of health care providers such as clinical pharmacists to contribute to a high-quality patient service. This stabilization profile is evidence of clinical contributions that can be repeated over time and are carried out by the same clinical group in the secondary care teams. This stabilization profile indicates that embedding dedicated practitioners within secondary care teams establishes reproducible clinical contributions that are sustained over time. This observed trend pattern closely mirrors longitudinal findings by other researchers following pharmacist integration in structured critical care in Riyadh facilities [12], which linked stable institutional documentation profiles directly to formal multidisciplinary round participation [19]. A retrospective eight-year Brazilian study conducted in an ICU further supports our findings, with over 96% intervention acceptance rate, linked to pharmacists’ presence in multidisciplinary rounds performed daily [20]. Al-Baha region trajectory demonstrates that peripheral health clusters can successfully maintain high-density clinical surveillance workflows once electronic tracking matrices and standardized practices are integrated.

The region had a high proportion of therapy optimization (28.4%) and dosage regimen corrections (23.6%), which is comparable to results from the large-scale national and global registries [6,12,13,15,20]. A retrospective study from a tertiary care hospital in western Saudi Arabia further confirmed that dosage errors constituted the largest single category of documented pharmacist interventions, accounting for 77.2% of all 38,143 recorded events across 2023 [12]. With multivariable logistic regression modeling, however, it is possible to identify predictor signals, which are not independent of each other. They are not visible in frequencies of description but give important information regarding operations. The strong positive association (OR 2.64, *p* < 0.001) with intensive care and negative association (OR 0.30, *p* < 0.001) with general wards strongly suggests a very distinct clinical specialization between the two settings. All critical care pharmacists provide real-time (bedside) physiological balancing, covering multi-organ support and a step-by-step antimicrobial de-escalation if needed. Clinical pharmacist-led antimicrobial stewardship programs have reported significant improvements in appropriate de-escalation for multidrug-resistant organisms, from 20% to 39.66% in a neurosurgical ICU and from 20.36% to 37.45% in a medical ICU, reinforcing the distinct specialized role of pharmacists in critical settings [21,22]. The ward practitioners, on the other hand, conduct entry-level prescription audits, discrepancy transcription and medication reconciliation when caring for patients in transitions of care. There is no time interval between the age ordinal band effect; it is independent and has a gerontological signal of therapy optimality. This finding’s profile is similar to others that identified patients with metabolic comorbidities or unique pharmacodynamic and pharmacokinetic profiles that can result in complex clinical scenarios requiring ongoing pharmacist care [23,24]. A prospective randomized clinical trial identified that a similar pharmacist-led medication reconciliation program reduced drug-related problems significantly in patients aged 65 years or above [25].

Among the 1077 active interventions with a documented physician decision across all three study years, 99.4% (1070 of 1077) were accepted or modified then accepted, indicating a high level of interprofessional communication and an established working relationship between clinical pharmacists and physicians in the Al-Baha MOH hospitals studied. Acceptance coding is documented across all three years, though completeness is lower for 2023 (94.4%) than for 2024 (97.8%) and 2025 (99.1%), consistent with the data-source change described in Section 2.2 and reported by others as well [14,15]. The high physician acceptance rate observed here is consistent with a recent review of acceptance rates globally, which found that clinical pharmacist integration into multidisciplinary teams substantially improves recommendation uptake and patient outcomes across hospital settings [26]. The acceptance rate is also corroborated by findings in a cardiovascular ICU in Thailand, where the pharmacist team documented high acceptance of both retrospective and prospective pharmaceutical interventions during ward rounds [27].

This is aligned with the target attainment rate for the INR of 74.5% recorded for the regional clinical ambulatory workforce, ensuring clinical credibility [28,29]. This baseline data could empower healthcare planners to make the case for increasing the involvement of pharmacists in the delivery of collaborative and outpatient medications and ensure they get scaled up where they are not currently available. These inputs obviously have direct links to the strategic plan of Saudi Vision 2030 and the health sector transformation program, providing a wider outlook on health policies. This work is even more important because it showed that meaningful interventions and adoption at the secondary level were possible even at high volumes. Al-Baha’s findings somewhat apply to the need to decentralize practitioner placement, as a large number of drug-related problems were observed. These outcomes reveal that there will be a demand from leadership of the health cluster at the regional level to invest at the regional level to build the infrastructure for pharmacies and establish more post-graduation residencies within the health cluster, as well as develop training modules for medication safety with an emphasis on a regional network to ensure the uniformity of the standards.

### 4.1. Implications for Practice, Strengthening Patient Safety Through Regional Pharmacist Integration

The findings carry direct relevance for patient safety and interprofessional practice in secondary care. A 99.4% physician acceptance rate indicates that clinical pharmacists in Al-Baha are functioning as trusted members of the ward team rather than as an external checking layer, a pattern associated with reduced prescribing error and improved hospital mortality outcomes [4]. The setting-dependent intervention profile, with ICU placement driving therapy optimization and general wards driving dosage-error correction, suggests that safety gains depend less on individual pharmacist skill and more on how pharmacist time is allocated across care settings. This has a practical staffing implication: regional health clusters seeking to reduce drug-related harm should prioritize embedding pharmacists at the point of highest risk, namely ICU and geriatric caseloads, rather than distributing coverage evenly [18]. High acceptance rates of this kind have also been linked internationally to formal inclusion of pharmacists in multidisciplinary ward rounds, and the Al-Baha data support extending that model of daily bedside participation to other peripheral Saudi facilities as a low-cost route to sustained intervention uptake [26].

The year-on-year shifts in Model 1 to 3 odds coincide with two changes in the study: a hospital-concentration shift confirmed directly in the dataset (see Limitations), and the 2023-to-2024 data-source change (Section 2.2). This shift is not evidenced by the physician-acceptance field, which is documented across all three years (94.4% of 2023 records, 97.8% of 2024, 99.1% of 2025), but by the change in mandatory fields and form structure between the 2023 regional registry and the 2024–2025 national platform described in Section 2.2. We do not attribute these shifts to system strain, software transition, or training lapses, as the dataset contains no variable that could test any of these hypotheses; the shift is reported descriptively.

### 4.2. Limitations

Several structural limitations apply to this investigation. First, the dataset contains a pharmacist identifier but no admission identifier, so the 1120 active interventions could not be reduced to unique admissions. All records are attributable to one of four named pharmacists, though two account for 99.5% of records (837 and 278) and the remaining two for 4 and 1 record, respectively, an imbalance as extreme as the hospital-level split described below, so pharmacist could not be modeled as a covariate or GEE clustering unit without risking near-complete separation for the two minority pharmacists. The chi-square analyses and the Ward cluster analysis therefore continue to treat each documented form as an independent observation, which may understate the true variance. The regression models address within-patient correlation directly, patient file numbers, recovered from the raw source files, allowed the complete-case regression sample to be clustered on patient identity (n = 1053 records, 433 clusters, 421 patients contributing more than one record, 12 singleton records) using GEE with an exchangeable working correlation structure and robust standard errors (Section 2.5), 42 of the 1120 active-intervention records (3.8%) had no recoverable file number and could not be linked to a patient. Clustering by hospital, pharmacist, or hospital admission could not be modeled, since no corresponding identifiers exist in the dataset. Second, data for 2023 were drawn from a regional administrative registry while 2024 to 2025 data came from a national electronic platform. A direct field-level comparison (Section 2.2) showed core analytical fields, including encounter category, are identically defined across both systems, and a sensitivity analysis restricted to the 346 of 372, 2023 active-intervention records with directly comparable intervention-type labels (excluding 26 records carrying 2023-specific labels with no 2024–2025 equivalent) gives an adjusted 2023 intervention rate of 45.5% (346/760) against the full-data 48.9%, the direction and magnitude of the 2023-to-2024 shift are unchanged. What cannot be determined from the files alone is whether mandatory field requirements, form sequencing, or documentation guidance differed between the two systems, so the rise in active-intervention rate cannot be attributed with confidence to programmatic integration, improved pharmacist activity, or a genuine change in drug-related problem burden, as opposed to a residual documentation-practice effect, and the years are therefore best interpreted with this caveat rather than as a fully continuous trend. Third, there is no direct measure of hard clinical outcomes such as mortality, readmission, or length of stay, so no claim of clinical benefit or harm reduction can be made from these data. Fourth, the two hospitals contributed active interventions in a strongly unbalanced ratio (99.6% Prince Meshari bin Saud Hospital versus 0.4% King Fahad Hospital), precluding hospital as a covariate in the regression models because of near-complete separation; the reported associations should be read as predominantly describing practice at the higher-volume site. Fifth, the Ward cluster analysis is reported as descriptive only, the underlying distance matrix (five features per intervention category—mean age band, ICU proportion, male proportion, and 2024 and 2025 proportions—with Euclidean distance and Ward’s minimum-variance linkage) has been re-verified for this revision and reproduces the same two-cluster split independently, but formal cluster-stability validation, such as testing alternative numbers of clusters or bootstrap resampling, was not performed. Sixth, several contingency tables involving rare intervention categories, such as Parenteral Nutrition (n = 5), or the strongly unbalanced hospital variable, had expected cell counts below 5, triggering chi-square approximation warnings in R; results for these sparser comparisons, particularly those involving hospital in the Cramér’s V matrix (Figure 7), should be interpreted with added caution. Further investigation should focus on prospective, multicentre studies across several peripheral health clusters, using patient-level identifiers and automated clinical pharmacy logs that also capture patient safety and health-economic outcomes.

## 5. Conclusions

This study provides early evidence describing clinical pharmacy services at the baseline level, through a three-year serial cross-sectional evaluation in the Al-Baha region. A large number of clinically relevant interventions were documented (mostly concentrated on intensive care and elderly patients), and this volume was consistent over time, the number of pharmacists contributing these records and their staffing levels were not captured in the dataset. In all multi-method analyses, clinical placement setting configuration and age of patients were found to be independent operational settings, and the latter led to different operational settings of patient profiles for interventions. However, it seems there has been a widespread shift, particularly when physicians are involved. Within the realm of clinical work, pharmacists in secondary care or specific regions have seemingly established a significant niche for themselves, becoming essential members of local teams. These results will be useful for regional health care planners and will help them continue investing in regional clinical pharmacy services in the Kingdom of Saudi Arabia and for workforce distribution planning and development of advanced educational programs in the region.

## Figures and Tables

**Table 1:** Patient demographics and clinical encounter configurations (n = 1120).

**Demographic and Operational Parameters**	**n (%)**
Total Documented Administrative Forms	1812 (100.0%)
Active Clinical Interventions	1120 (61.8%)
File Reviews, No DRP Identified	145 (8.0%)
Ambulatory Anticoagulation Follow-ups	543 (30.0%)
Medication reconciliation forms	4(0.2%)
**Total** ** ** **Documented** ** ** **records** ** ** **by** ** ** **Study** ** ** **Year**	**n (%)**
2023	760 (41.9%)
2024	568 (31.3%)
2025	484 (26.7%)
**Gender** ** ** **Spectrum** ** ** **Distribution**	
Male Patients	589 (52.6%)
Female Patients	479 (42.8%)
Unrecorded Registry Entries	52 (4.6%)
**Age** ** ** **Stratification** ** ** **Cohorts**	
Pediatric and Young Adults (0–24 years)	64 (5.7%)
Adult and Mature Cohorts (25–54 years)	209 (18.7%)
Pre-Geriatric Cohort (55–64 years)	122 (10.9%)
Geriatric Cohort (65 years and above)	684 (61.1%)
System missing (Undocumented)	41 (3.7%)

Values are n (%). DRP = Drug-related problem. A further 4 records (0.2%) were medication reconciliation forms, not itemized as a separate row above, 145 + 543 + 4 = 692 non-active forms.

**Table 2:** Distribution of clinical pharmacist intervention domains stratified by study year (n = 1120).

Intervention Domain Classification	Total Interventions, N (%)	Baseline Year 2023, n (%)	Interim Year 2024, n (%)	Contemporary Year 2025, n (%)
Therapy Optimization	318 (28.4%)	137 (36.8%)	104 (25.3%)	77 (22.8%)
Dosage Regimen Error	264 (23.6%)	38 (10.2%)	121 (29.4%)	105 (31.2%)
Missing Drug Order	210 (18.8%)	58 (15.6%)	78 (19.0%)	74 (22.0%)
Electrolyte Correction	121 (10.8%)	55 (14.8%)	43 (10.5%)	23 (6.8%)
ADR/Drug Interaction	47 (4.2%)	14 (3.8%)	18 (4.4%)	15 (4.5%)
Inappropriate Drug Selection	39 (3.5%)	16 (4.3%)	18 (4.4%)	5 (1.5%)
No Indication	38 (3.4%)	12 (3.2%)	7 (1.7%)	19 (5.6%)
Drug Unavailability	32 (2.9%)	14 (3.8%)	6 (1.5%)	12 (3.6%)
Patient Education	28 (2.5%)	16 (4.3%)	9 (2.2%)	3 (0.9%)
TDM/Monitoring	18 (1.6%)	7 (1.9%)	7 (1.7%)	4 (1.2%)
Parenteral Nutrition Support	5 (0.4%)	5 (1.3%)	0 (0.0%)	0 (0.0%)
Total	1120 (100.0%)	372 (100.0%)	411 (100.0%)	337 (100.0%)

Data values are presented as absolute frequency (n) followed by percentage (%). ADR = Adverse Drug Reaction. TDM = Therapeutic Drug Monitoring.

**Table 3:** Distribution of pharmacist interventions by clinical specialty setting (n = 1120).

Clinical Placement Setting	n (%)
Adult Intensive Care Unit (ICU)	830 (74.1%)
Adult Internal Medicine Wards	279 (24.9%)
Ambulatory Anticoagulation Clinic	4 (0.4%)
Neonatal Intensive Care Unit (NICU)	3 (0.3%)
Institutional Surgical Wards	2 (0.2%)
Pediatric Critical Care and Cardiology	2 (0.2%)

Data reflect localized distributions across clinical wards within the regional cluster ledger, formatted as a unified absolute frequency (n) and percentage (%).

**Table 4:** Inferential testing framework and nominal association strength matrix.

Association	Test	Statistic	Cramer’s V/Effect Size	Significance (*p*-Value)
Intervention Domain vs. Clinical Setting	Chi-square	203.43 (df = 10)	0.426	<0.001
Intervention Domain vs. Patient Gender	Chi-square	18.69 (df = 10)	0.132	0.044
Intervention Domain vs. Age Cohorts	Chi-square	16.36 (df = 10)	0.123	0.090 (NS)
Intervention Domain vs. Observation Year	Chi-square	108.86 (df = 20)	0.220	<0.001
Intervention Volume Growth, 2023 vs. 2024	Two-Prop. Z-test	−8.581	-	<0.001
Intervention Volume Shift, 2023 vs. 2025	Two-Prop. Z-test	−7.183	-	<0.001
Intervention Volume Stability, 2024 vs. 2025	Two-Prop. Z-test	0.974	-	0.330 (NS)

df = degrees of freedom, NS = not significant. Cramér’s V interpreted per Section 2.5 (moderate = 0.30–0.49). Significant entries, *p* < 0.05.

**Table 5:** Multivariable logistic regression models predicting independent drivers of pharmacist interventions.

Predictor Covariates	Model 1, ICU Placement aOR [95% CI]	Model 1 (*p*)	Model 2, Dosage Error aOR [95% CI]	Model 2 (*p*)	Model 3, Therapy Optimization aOR [95% CI]	Model 3 (*p*)
Age Band (Ordinal)	1.162 [1.010–1.337]	0.036	0.974 [0.877–1.082]	0.622	1.078 [0.986–1.179]	0.098
Male (vs. Female)	0.919 [0.695–1.215]	0.554	1.059 [0.772–1.450]	0.721	0.894 [0.676–1.183]	0.432
ICU Setting	Not Included	—	0.303 [0.213–0.431]	<0.001	2.637 [1.791–3.882]	<0.001
Year 2024 (vs. 2023)	0.092 [0.044–0.191]	<0.001	2.572 [1.651–4.007]	<0.001	0.661 [0.465–0.940]	0.021
Year 2025 (vs. 2023)	0.029 [0.014–0.062]	<0.001	1.878 [1.119–3.151]	0.017	0.747 [0.515–1.082]	0.122

aOR = adjusted odds ratio (GEE, exchangeable working correlation, robust standard errors), CI = confidence interval. Reference groups: female gender, year 2023. Clustering unit: patient file number. n = 1053 records, 433 clusters (421 patients with a recoverable file number, 12 singleton records without one). Model 1 predictors: age band, gender, year. Models 2–3 predictors: age band, gender, ICU setting, year.

## Data Availability

The Raw data supporting the conclusions of this study will be made available by the authors upon request.

## Supplementary Materials

The following supporting information can be downloaded at: https://www.mdpi.com/article/10.3390/healthcare14172689/s1, Table S1: Classification rules. Table S2. Year by hospital by clinical setting cross-tabulation, active interventions (n = 1120). Table S3. Pharmacist by year, active interventions (n = 1120). Figure S1. Data flow diagram: extraction, per-source training/lecture exclusion, concatenation, deduplication check, and allocation to encounter category (Methods Section 2.3).

## Abbreviations

The following abbreviations are used in this manuscript,

MOH	Ministry of Health
DRP	Drug-related problem
ICU	Intensive Care Unit
NICU	Neonatal Intensive Care Unit
PICU	Pediatric Intensive Care Unit
LOS	Length of stay
NTI	Narrow-therapeutic-index
ADR	Adverse Drug Reaction
DI	Drug Interaction
TDM	Therapeutic Drug Monitoring
OR	Odds ratio
aOR	Adjusted odds ratio
CI	Confidence interval
LR	Likelihood ratio
INR	International normalized ratio
