# Supplementary material for: Evaluating Clinical Pharmacy Services in Ministry of Health Hospitals in Al-Baha Region of Saudi Arabia, a Three-Year Retrospective Analysis
Source: Healthcare (Basel). 2026 Aug 24;14(17):2689. doi: 10.3390/healthcare14172689 (PMC13565221; doi:10.3390/healthcare14172689)
Supplement: Supplementary file 1 [file healthcare-14-02689-s001.zip › healthcare-4477685-supplementary.pdf]

**Supplementary Data**

**Methods:**

**Data Flow and Record Selection**

Records were extracted from three source files covering the Al Baha region: a 2023 regional file (831 rows), a 2024–2025 regional file (861 rows), and a national dataset (71,242 rows spanning 20 regions). The national dataset was first filtered to Al Baha records (n = 1,108), from which 861 records already present in the regional 2024–2025 file were identified by exact timestamp matching and removed, yielding 247 unique 2025 records not otherwise captured. All three sources were then concatenated and deduplicated by timestamp, producing 1,812 intervention form records. Each record in the MOH digital platform is generated by one of three form types: intervention forms documenting direct patient care activity, training forms recording pharmacist education events, and lecture forms recording educational sessions. Training forms and lecture forms were identified by the platform-assigned form-type field and excluded within each source file before concatenation: 54 training and 17 lecture records from the 2023 regional file (831 to 760), 34 training and 9 lecture records from the 2024-2025 regional file (861 to 818), and 6 training and 7 lecture records from the unique 2025 subset of the national file (247 to 234), a total of 94 training and 33 lecture records excluded. The three cleaned sources were concatenated (760 + 818 + 234 = 1,812) and checked for exact-timestamp duplicates, of which none were found. The complete extraction and exclusion pipeline is shown in Supplementary Figure S1. Within the 1,812 patient-care records, the platform's category-of-intervention field identified four encounter types: active pharmacist interventions (n = 1,120), file reviews in which no drug-related problem was identified (n = 145), anticoagulant clinic and outpatient ambulatory follow-ups (n = 543), and medication reconciliation visits (n = 4). The primary analysis group comprised the 1,120 active intervention records. Sample sizes for each regression model reflect records with complete data on all covariates and are reported in Table 5; 41 records (3.7%) had no age documented in the source system and were excluded from age-stratified and regression analyses.

**Intervention Type Classification**

Intervention type was classified using automated keyword matching applied to the raw free-text strings entered by pharmacists into the MOH digital platform. Classification was performed computationally, without manual review of individual records and without a second independent coder. A deterministic, priority-ordered ruleset of 11 patterns was applied case-insensitively to each record; the first matching rule determined category assignment, ensuring mutual exclusivity. The full ruleset, including the regular-expression pattern for each category and the rationale for its formulation, is provided as Supplementary Table S1. No inter-rater reliability coefficient was calculated, as there

was no human coding process against which to assess agreement. Where a single consultation form contained documentation of more than one clinical issue, the platform captured only the primary intervention type in the field analysed; secondary issues recorded within free-text narrative fields were not extracted for categorical analysis. Records matching no rule were assigned to an "Other" category for manual review; no such records were identified in the present dataset.

Platform terminology evolved between 2023 and 2024 following a system update, and several labels used in an earlier draft of this manuscript did not correspond to the platform-assigned category names used in Table 2. These discrepancies are resolved as follows. The term "guideline deviation" used in the earlier Methods draft refers to the platform category Inappropriate Drug Selection, defined by the platform label "inappropriate drug selection against guideline or recommendation." The term "failure to order an electrolyte" used in the earlier draft corresponds to the platform category Electrolyte Correction, labelled "correct the cause of electrolyte disturbance." The term "structured patient counselling" corresponds to Patient Education, labelled "providing information: counselling or education." The category Parenteral Nutrition, labelled "parenteral nutrition support," was inadvertently omitted from the earlier Methods list and is included in the corrected ruleset; it accounted for five records (0.4%) across the study period. All category labels used in Table 2 reflect platform terminology and are mapped explicitly to the classification rules in Supplementary Table S1. Python v3.13 code was used to perform the job.

<table>
<tr><th>Priority</th><th>Category_Assigned</th><th>Regex_Pattern</th><th>Rationale_and_Platform_Label</th></tr>
<tr><td>1</td><td>Therapy Optimisation</td><td>optim</td><td>Matched on "Optim" (platform label: "Optimize the patient therapy"); includes antibiotic de-escalation, dose optimisation, guideline-directed initiation of evidence-based therapy</td></tr>
<tr><td>2</td><td>Dosage Regimen Error</td><td>dosage|inappropriate dosage|inappropriate regimen</td><td>Matched on "dosage" or "inappropriate dosage regimen" (platform label); sub-types (route, dose, frequency, duration) captured in separate column "Inappropriate dosage regimen (details)"</td></tr>
<tr><td>3</td><td>Missing Drug Order</td><td>no drug order|missing.*drug|drug.*order.*medical condition</td><td>Matched on "no drug order for medical condition" (platform label); covers untreated clinical conditions and omitted indicated medications</td></tr>
<tr><td>4</td><td>Electrolyte Correction</td><td>electrolyte|correct the cause|electrolyte disturbance</td><td>Matched on "electrolyte" or "correct the cause of electrolyte disturbance" (platform label); reviewers note: labelled "failure to order an electrolyte" in Methods draft â€” corrected here to match platform terminology</td></tr>
<tr><td>5</td><td>ADR/Drug Interaction</td><td>adr|adverse drug|experiencing.*adr|drug interaction|side effect|contraindication|toxicity</td><td>Matched on "ADR", "experiencing ADR", "drug interaction", "contraindication", "toxicity" (platform label: "Experiencing ADR/Side effect/Contraindication/Toxicity/Drug interaction")</td></tr>
<tr><td>6</td><td>Inappropriate Drug Selection</td><td>drug selection.*guideline|against guideline|inappropriate.*selection|guideline.*recommendation</td><td>Matched on "drug selection Against guideline/recommendation" (platform label); includes guideline deviation â€” the Methods term "guideline deviation" maps to this category</td></tr>
<tr><td>7</td><td>Patient Education</td><td>providing information|counselling|counselling|patient education|education</td><td>Matched on "Providing information (counseling/education)" (platform label); reviewers note: described as "structured patient counselling" in Methods draft â€” corrected here to match platform terminology</td></tr>
<tr><td>8</td><td>TDM/Monitoring</td><td>tdm|therapeutic drug monitoring|monitoring drug effect|drug.*monitor</td><td>Matched on "TDM" or "Monitoring drug effect or side effect/TDM" (platform label)</td></tr>
<tr><td>9</td><td>Drug Unavailability</td><td>unavailab|non formulary|alternative.*medication.*unavail|recommend alternative</td><td>Matched on "unavailability", "non formulary", "recommend alternative medication in case of unavailability" (platform label)</td></tr>
<tr><td>10</td><td>No Indication</td><td>no indication|no drug indication</td><td>Matched on "No indication for drug therapy" (platform label)</td></tr>
<tr><td>11</td><td>Parenteral Nutrition</td><td>parenteral|parenteral nutrition|pn support|total parenteral</td><td>Matched on "parenteral" or "PN"; this category appears in Table 2 but was inadvertently omitted from the Methods classification list â€” added here</td></tr>
</table>

Supplementary Table S1: Classification rules

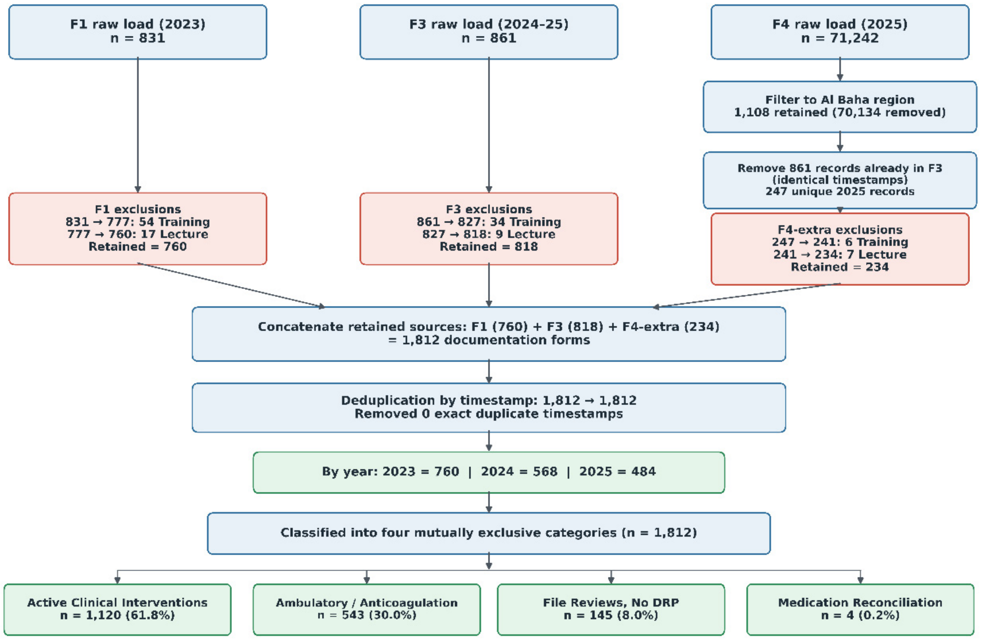


**Supplementary Figure S1.** Data flow diagram: extraction, per-source training/lecture exclusion, concatenation, deduplication check, and allocation to encounter category (Methods Section 2.3).

**Supplementary Table S2.** Year by hospital by clinical setting cross-tabulation, active interventions (n = 1,120)

| Year | Hospital | Clinical Setting | N (% within year) |
|---|---|---|---|
| 2023 | KF_AlBaha | Anticoagulant clinic | 4 (1.1%) |
| 2023 | PMSB_Baljurashi | Cardiology | 1 (0.3%) |
| 2023 | PMSB_Baljurashi | ICU | 363 (97.6%) |
| 2023 | PMSB_Baljurashi | NICU | 3 (0.8%) |
| 2023 | PMSB_Baljurashi | Surgical | 1 (0.3%) |
| 2024 | PMSB_Baljurashi | ICU | 303 (73.7%) |
| 2024 | PMSB_Baljurashi | Internal Medicine | 108 (26.3%) |
| 2025 | PMSB_Baljurashi | ICU | 164 (48.7%) |
| 2025 | PMSB_Baljurashi | Internal Medicine | 171 (50.7%) |
| 2025 | PMSB_Baljurashi | PICU | 1 (0.3%) |
| 2025 | PMSB_Baljurashi | Surgical | 1 (0.3%) |

*99.6% of active-intervention records originate from Prince Meshari bin Saud Hospital (PMSB_Baljurashi); King Fahad Hospital (KF_AlBaha) contributed 4 records, all in the 2023 anticoagulant clinic. An Internal Medicine setting appears only from 2024 onward, coincident with the change in data-source system described in Methods Section 2.2, and is addressed in the Limitations.*

**Supplementary Table S3.** Pharmacist by year, active interventions (n = 1,120)

| Pharmacist | 2023 | 2024 | 2025 | Total | % of 1,120 |
|---|---|---|---|---|---|
| Dr. Ali Serhan Alghamdi | 367 | 303 | 167 | 837 | 74.7% |
| Dr. Mohammed Salem Alghamdi | 0 | 108 | 170 | 278 | 24.8% |
| Dr. Abdulhakim Ali Alzahrani | 4 | 0 | 0 | 4 | 0.4% |
| Dr. Lujin Khayyat | 1 | 0 | 0 | 1 | 0.1% |

*All 1,120 active-intervention records carry a documented pharmacist identifier (Methods Section 2.1, Limitations). All four pharmacists practiced at Prince Meshari bin Saud Hospital except Dr. Abdulhakim Ali Alzahrani, whose 4 records are the entirety of King Fahad Hospital's active-intervention contribution described in Supplementary Table S2. Two pharmacists account for 99.5% of records (837 and 278); the remaining two contributed 4 and 1 record respectively, an imbalance comparable to the hospital-level concentration in Supplementary Table S2, which is why pharmacist was not modelled as a covariate or GEE clustering unit. Dr. Mohammed Salem Alghamdi's records begin only in 2024, coincident with the 2023-to-2024 platform change described in Methods Section 2.2, whether this reflects a genuine staffing addition or a documentation-system artefact cannot be determined from the files alone.*
